# Brain criticality and advanced meditation “cessation” events: An intensively sampled electroencephalography case study

**DOI:** 10.1016/j.ibneur.2026.07.011

**Published:** 2026-07-25

**Authors:** Remko van Lutterveld, Thomas Cahaly, Lukas van Herk, Daniel M. Ingram, Matthew D. Sacchet

**Affiliations:** a Brain Research and Innovation Centre and Department of Psychiatry, Ministry of Defence and University Medical Center, Utrecht, the Netherlands; b Meditation Research Program, Department of Psychiatry, Massachusetts General Hospital, Harvard Medical School, Boston, MA, USA; c Emergent Phenomena Research Consortium, New Market, AL, USA

**Keywords:** Cessation, Advanced Meditation, Nirodha, Mindfulness, Detrended Fluctuation Analysis, Critical Point

## Abstract

Advanced meditation includes meditative endpoints that are outcomes of meditation practice and occur with time and mastery of meditation. The “cessation” event (c.f. *nirodha* in Theravada Buddhism) is a meditative endpoint characterized by a loss of consciousness followed by a sense of mental reset and deep clarity. In general, organization of brain activity exists along a “criticality” spectrum: order and disorder occupy the extremes, and the critical point, which is thought to represent optimal information processing, is in between. In this exploratory, hypothesis-generating case study, we assessed brain criticality in the 39.9 s pre-cessation to 39.9 s post-cessation window by applying detrended fluctuation analysis (DFA) to electroencephalography (EEG) data in relation to 37 cessations in a single advanced meditator. Changepoint analysis and linear mixed effects modeling revealed a spike in the whole-brain DFA exponent starting from 11.3 to 3.1 s pre-cessation, suggesting a strengthening of long-range temporal correlations (LRTC) and movement towards the critical point. In addition, a sustained elevation was observed from 3.1 to 23.6 s post-cessation. Linear changepoint analysis effects were most pronounced in central, right frontal, and posterior EEG channels, while frontal channels Fp1, F3, and F7 incurred into supercritical territory surrounding cessations. Additional analysis revealed a temporary drop in the mean DFA exponent around cessation onset relative to immediately surrounding segments. For most channels, the mean DFA exponent reached a time series maximum at 1.0–3.1 s following cessation onset, often near the critical point or just into the supercritical range. These preliminary findings in a single case study, which are consistent with reported phenomenology of cessations, show large-scale modulation in criticality related to cessations.

## Highlights

• Brain criticality was assessed before and after advanced meditation cessation events.
• Brain criticality spiked pre-cessation.
• Linear effects were most prominent in central, posterior, and right frontal channels.
• Near-critical and supercritical dynamics were observed around and during cessations..

## 1 Introduction

Advanced meditation encompasses an array of states, stages, and practices that are experienced through time and mastery of meditative techniques (Sacchet et al. 2024; Winson Fu Zun Yang et al. 2024a; Shinozuka et al. 2025; W.F. Yang, Potash, et al. 2025; Sacchet and Lieberman, 2026; Tal, Yang, et al. 2026; Zarka et al. 2026). Unsurprisingly, there is growing interest in the study of advanced meditation (Wright et al., 2023, Sacchet et al., 2024) and development of the methods used to study it (Nath et al., 2025, Grabovac et al., 2026, Kumar et al., 2026), with a new focus of research in contemplative science that has been termed the ‘third wave of meditation research’ (Sacchet et al., 2024, Sparby and Sacchet, 2025a, Murray et al., 2026). Advanced meditation includes the unfolding of stages of meditation and what we have called ‘meditative development’ (Cain et al., 2024, Ehmann and Lord, 2025, Ehmann and Sezer, 2025, Ehmann et al., 2025, Galante et al., 2023, Sparby and Sacchet, 2025b), that ultimately leads to meditative endpoints (Chowdhury et al., 2023, van Lutterveld et al., 2024, Sparby and Sacchet, 2025). Previous meditation research has provided various neuroscientific insights on topics, such as brain connectivity (Sezer et al., 2022, van Lutterveld et al., 2024, Potash et al., 2024, Demir et al., 2025, Prakash et al., 2025; W.F. Yang, Kadambi, et al. 2025), mental and physical health (Winson F.Z. Yang et al. 2024b; Ehmann, Sparby, Naughton, et al. 2025; Ganesan et al. 2025; Prueitt et al. 2025; Sezer and Sacchet, 2025; Hugemark et al. 2026), and the conscious human experience in general (Abellaneda-Pérez et al., 2024, Ehmann et al., 2024, Bonamino et al., 2026). Moreover, such research complements studies on other subjects, including emergent phenomena (Wright et al., 2024, Wright et al., 2025), brain stimulation (Rayani et al. 2025), and psychedelics (McAlpine et al., 2025, McAlpine et al., 2026, Riedel et al., 2026).

These meditative states and endpoints can be described and classified using phenomenology (Sparby and Sacchet, 2022, Sparby and Sacchet, 2024, Tal and Wright, 2026). One particular meditative endpoint is a “cessation” event (c.f. evolving definitions of *nirodha* in Pali as conceived by various sects and across different eras within Theravada Buddhism). These events are characterized by a brief loss of consciousness that usually last a few seconds (Sayadaw, 1994, Ñāṇamoli, 1995). As described by practitioners, one may emerge from a cessation event with a feeling of deep bliss and that something fundamental within them has reset or changed (Grabovac, 2015).

There exists a research interest in cessations to not only explore the neuroscience of the events themselves, but also better understand the limits of meditation and human consciousness more broadly. Three previous papers have conducted neuroscientific investigation of cessation events. Berkovich-Ohana (2017) analyzed a total of six cessations in two participants and found an increase in global long-range gamma synchronization during these events (please note that these authors refer to cessations as “fruitions”). Next were two intensively sampled case studies that used the same dataset as the present paper. First, Chowdhury et al. (2023) showed a large-scale linear decrease in alpha-power and modest linear theta-power increase leading up to cessation with a linear recovery in alpha power post-cessation. Additionally, van Lutterveld et al. (2024) demonstrated a linear decrease in large-scale whole-brain functional interactions in the alpha band pre-cessation, and then a linear recovery to baseline after cessation. Although these studies provide evidence regarding power and functional connectivity neural correlates surrounding cessation events, they do not provide information regarding critical dynamics of the brain.

In general terms, a system is operating at the critical point or within the critical region if it is balanced at the phase transition between order and disorder (Hesse and Gross, 2014). Many studies show that a healthy brain operates near this point; closeness to criticality is associated with increased long-range temporal correlations (LRTC) and optimized information processing (Beggs and Plenz, 2003, Beggs, 2008, Priesemann, 2014, Ma et al., 2019, O’Byrne and Jerbi, 2022). Indeed, various disorders such as schizophrenia and Alzheimer’s disease are associated with departures from criticality (Zimmern, 2020), and there is some evidence that departures from criticality are predictive of future psychotherapy treatment success (Van Lutterveld et al. 2025). Given these associations, interest in brain criticality has expanded greatly in recent years. Previous studies have investigated critical dynamics during focused attention meditation practice (Irrmischer et al., 2018, Walter and Hinterberger, 2022a, D’Andrea et al., 2024), observing a weakening of long-range temporal correlations and thus a movement away from the critical point. Critical brain dynamics have also been proposed to underlie consciousness more broadly (Walter and Hinterberger 2022b), and are for example linked to anesthesia-induced unconsciousness (Thiery et al. 2018), which supports the broader idea that critical dynamics vary depending on the cognitive state (O’Byrne and Jerbi, 2022). Because cessations are marked by a loss of consciousness and change in cognitive state, a link between critical dynamics and cessation events is expected. Indeed, a recent review states that “criticality could become, with more extensive research, an effective and objective way to characterize altered states of consciousness” (Gervais et al., 2023). However, limited research has investigated criticality related to advanced meditation or meditative endpoints (Vohryzek et al. 2025).

In the present exploratory, hypothesis-generating study, we aimed to address this gap in the literature and used an intensive sampling case study design focusing on a single advanced meditator capable of cessations during their meditation practice. Intensive case study designs have been extremely valuable in previous meditation research, especially when studies focus on rare skill sets (Ganesan et al. 2024; Winson Fu Zun Yang et al. 2024b; Chowdhury et al. 2025; Potash et al. 2025; Treves et al. 2025; Yang, Chowdhury, et al. 2025). Using the same rare and intensive dataset as Chowdhury et al. (2023) and van Lutterveld et al. (2024), we performed detrended fluctuation analysis (DFA), a common criticality analysis approach, to assess critical dynamics surrounding cessation events recorded using electroencephalography (EEG). Specifically, we computed the DFA exponent, which is used to characterize the strength of long-range temporal correlations. Thiery et al. (2018) found an increase in the DFA exponent towards a value of one during anesthesia-induced unconsciousness, which represents a movement towards the critical point (Hardstone et al., 2012, O’Byrne and Jerbi, 2022). Therefore, surrounding cessation events we hypothesize an increase in the DFA exponent towards a value of one and a movement towards brain criticality.

## 2 Materials and methods

### 2.1 Participant

The singular case study participant was co-author DMI, who is a male advanced meditator and teacher with 26 years of meditation experience at the time of data acquisition. He had an estimated lifetime practice duration of 23,110 h (Chowdhury et al. 2023), with over 6000 estimated hours spent in meditation retreats (van Lutterveld et al. 2024). His practice included Mahasi noting, formed and formless jhanas, kasinas, and Vipassana (or Insight) meditation. He was 50–51 years old during data collection. This study was approved by the Western Institutional Review Board (WIRB) and the participant provided informed consent.

### 2.2 EEG

#### 2.2.1 Data acquisition

Data acquisition and selection was performed identical to Chowdhury et al. (2023) and van Lutterveld et al. (2024). First, data were acquired using a Quick−20 EEG system with 19 dry electrodes positioned according to the 10–20 system (Cognionics, San Diego, CA, USA). The sampling frequency was 500 Hz. The participant performed one of two meditation practices with eyes closed: either “review practice” Vipassana meditation, where the participant inclined towards cessation, or fire kasina, a traditional Theravada practice which involves internally-generated visual sensations (Ñāṇamoli, 2010). Vipassana meditation was performed for 16 runs, while fire kasina meditation was performed for 13 runs. In total, 29 EEG runs were recorded, ranging from 426 to 3653 seconds. During some runs, multiple cessations occurred.

**Fig. 1:**
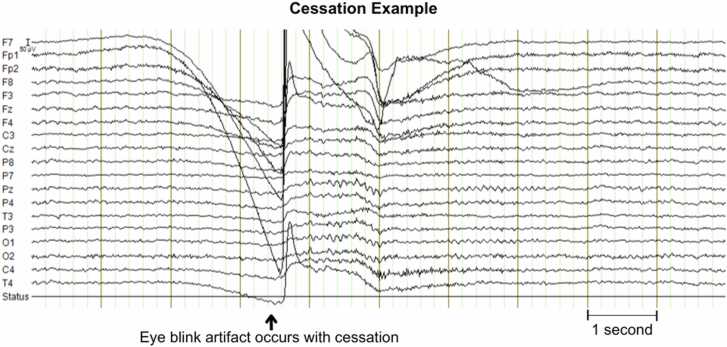
EEG recording of cessation event and associated involuntary eye blink artifact, which was excluded from analysis. Figure adjusted from Chowdhury et al. (2023).

Cessation events were identified by involuntary motion artifacts that occurred alongside cessations (a large eye blink accompanied by eye fluttering or sometimes squinting). These eye blinks are visually obvious on the EEG recording and thus easily identified (see Fig. 1 for an example). The accuracy of identified cessation events was validated in two ways. First, the participant and a co-author adept in EEG analysis independently reviewed the EEG runs and marked the same locations for involuntary eye blink events. Moreover, in one run which contained seven cessations, cessations were additionally marked by button press directly after the event by the participant. The median time between the cessation-related eye blink artifact onset and button press was 3.6 s (ranging from 2.1 s - 7.2 s) (Chowdhury et al. 2023). These events were therefore short and in accordance with the literature (Sayadaw, 1994, Laukkonen et al., 2023). To reduce confounding monitoring processes button-presses were excluded from the other runs (van Lutterveld et al. 2013).

Directly following each run, the participant graded the quality of the cessation events as either Grade A, Grade B, or Grade C based on the clearness and correctness of several aspects of the cessation: setup, entrance, the cessation itself, exit, and afterglow. In a Grade A cessation, all factors were clear and correct, while a Grade B rating featured one unclear factor. Finally, a Grade C rating meant multiple unclear factors. Supplementary Material Text S1 provides additional grading details, identical to Chowdhury et al. (2023). Across all EEG runs, 46 Grade A, 12 Grade B, and 11 Grade C cessations occurred. We focused on Grade A cessations for further analysis as these provided the largest statistical power and most likely presented the purest form of cessation events. To reduce carry-over effects from adjacent cessations, additional criteria for inclusion were that cessations occurred at least 1) 80 s from other cessations, 2) 40 s after the start of the EEG run, and 3) 40 s before the end of the EEG run. One dataset was excluded from analysis because of technical difficulties. After excluding cessations that did not meet these criteria, 37 of the 46 Grade A cessations were selected for further analysis. To enable a temporal control analysis (in which data surrounding cessations can be assessed versus data temporally removed from cessations in the same data sets), control markers were inserted. First, time segments were identified that were at least 1) 160 s from a cessation event to mitigate the impact of any residual effects, 2) 40 s after the start of the EEG run, and 3) 40 s before the end of the EEG run. All segments of EEG runs that were long enough and fulfilled these criteria were identified, and a control marker was randomly placed in each of these segments. In total, 27 control runs of the same length as cessation runs were isolated for further analysis.

#### 2.2.2 Preprocessing

Similar to van Lutterveld et al. (2024), preprocessing was performed using the BrainVision Analyzer suite (BrainProducts, Munich, Germany). Bad channels were identified using visual inspection and subsequently removed from analysis (median = 0, range = 0–3). A 0.5 Hz to 100 Hz Butterworth Infinite Impulse Response (IIR) filter with a slope of 48 dB/octave was applied. EEG data was segmented into 39 segments of 1024 samples covering the 39.936 s pre-cessation or pre-control marker to 39.934 s post-cessation or post-control marker time series. Given the sampling frequency of 500 Hz, each segment represented 2.048 s. Automated artifact identification was performed using 50 µV/ms as the maximum allowed voltage step, 200 µV as the maximum allowed amplitude difference in 200 ms intervals, ±100 µV as the maximum and minimum allowed amplitudes, and 0.5 µV as the lowest allowed activity in 100 ms intervals. Furthermore, all data were visually inspected by an experienced EEG scientist (RvL) to assess artifacts that were not picked up by the automated pipeline. These were mainly motion/muscle activity. Segments containing undetected artifacts were removed from analysis. Due to the involuntary artifact that occurred with the cessation, the middle segment containing the cessation as well as the segments directly before and after (segments 19–21, corresponding to 3.1 s pre-cessation to 3.1 s post-cessation) were not included in the main analysis. Due to electromyogram (EMG) contamination in EEG frequencies above 20 Hz, additional frequency filtering was performed (Whitham et al., 2007, Pope et al., 2022). The EEGLAB software (Delorme and Makeig, 2004) within MATLAB R2023A (MathWorks, Natick, MA, USA) was utilized to perform a fast Fourier transform (FFT) linear filter in the 1–20 Hz range. The order of the filter was automatically determined by the software based on the sampling rate; per software recommendations, high- and low-pass filtering were conducted separately. The low-density EEG cap used for data collection limits Independent Component Analysis, which was therefore not performed (Lau et al. 2012).

### 2.3 Detrended fluctuation analysis

DFA, a method developed by Peng et al. (1994), is used to characterize the self-affinity of a time series signal. That is, DFA measures the long-term memory of time series. In a self-affine time series, smaller parts of the signal display trend similarities to the entire signal. The “scaling” or “DFA” exponent α, an estimation of the Hurst Exponent H, is the ultimate result of DFA. This value provides insights into how the signal is changing over time. For 0<α<0.5 the signal is stationary, has a memory, and is anticorrelated, while α=0.5 indicates that the signal is random white noise. In contrast, for 0.5<α<1, the signal is stationary, has a memory, but is correlated, which is a hallmark of operation near the critical point (Zimmern, 2020). A signal is anticorrelated if it generally behaves differently to previous actions and correlated if it generally behaves in a similar manner to previous actions. As the DFA exponent approaches 1, long-range temporal correlations become stronger, indicating the signal is approaching the critical point (Hardstone et al., 2012, O’Byrne and Jerbi, 2022) and at a higher risk of sudden large changes (Kroha and Škoula, 2018). Finally, the signal is non-stationary if 1<α<2 (Hardstone et al. 2012), meaning there is a shift in mean or variance over the time series. Please see Supplementary Material Text S2 for an overview of the DFA procedure.

DFA analysis was performed using the DFA toolbox DFluC (Colombo et al. 2016) within MATLAB. By default, this program automatically divides the time series into 50 boxes and uses a second-degree polynomial for local detrending (Walter and Hinterberger 2022a). The smallest box size was increased from 51 (default) to 67 to reduce crossover in the resulting log-log plots that the DFA exponents were derived from. Previous research suggests that the resulting DFA exponent is similar when using both a second-degree and a higher order polynomial when the time series length exceeds 1000, which is the case in the present study (Zhao et al. 2022). However, DFA can also be utilized successfully for time series of 1000 samples and shorter (Delignieres et al., 2006, Kirchner et al., 2014, Kuznetsov and Rhea, 2017), including with EEG data (Wairagkar et al. 2021). We analyzed broadband DFA, which was the focus in many previous EEG DFA studies (e.g., Hou et al. 2017; Wairagkar et al. 2021), including meditation research (Walter and Hinterberger 2022a). DFA was run on each EEG channel time series for all cessation and control runs. In the main analysis, for each cessation and control segment (in total covering the 39.9–3.1 s pre-cessation or pre-control marker and 3.1–39.9 s post-cessation or post-control marker timeframes), a whole-brain average DFA exponent was calculated by averaging the mean DFA exponents for that segment across all 19 channels. Mean DFA exponents for each segment per channel were also assessed.

### 2.4 Statistical analysis

Modulation of brain criticality was statistically assessed in two ways. First, changepoint analysis was performed to assess whether there are pre- or post-cessation time-points where DFA exponents changed linear trends over time. Second, pre- and post-cessation DFA exponents were tested versus temporal control DFA exponents using linear mixed effects modeling. All statistical analysis was performed within MATLAB or RStudio/R.

#### 2.4.1 Changepoint analysis

The Bayesian Estimator of Abrupt change, Seasonal change, and Trend (BEAST) toolbox was utilized to detect changepoints (Zhao et al. 2019). In contrast to other algorithms that provide one best model, BEAST is a “fuzzy” algorithm that uses Bayesian model averaging to determine probabilities of not only the total number of changepoints in a time series, but also the likelihood of each individual point being a changepoint. BEAST has been used successfully to study many disciplines, such as ecology, ichthyology and hydraulic engineering (Adams et al., 2021, Xu et al., 2022, Kaeding, 2023). For a complete technical explanation please consult Zhao et al. (2019). We ran BEAST separately on the pre-cessation, post-cessation, pre-control marker, and post-control marker time series. For settings, seasonality was disregarded, as there is no expected periodic component in our time series. Otherwise, default parameters were used: zero and one order polynomials (straight lines) were allowed to be considered by the algorithm to detect changepoints. This testing for linear effects was informed by prior studies showing linear effects leading up to and following cessations (Chowdhury et al., 2023, van Lutterveld et al., 2024). A minimum 50% probability threshold for both the mode (or total number of changepoints) and individual changepoint probabilities were utilized to identify significant changepoints. These cutoffs are in line with previous literature (Mulverhill et al., 2023, Ganji et al., 2024, Stamieszkin et al., 2024). This means, for instance, to consider two changepoints in a time series, there must be at least a 50% probability that there is a total of two changepoints as well as at least a 50% probability for each individual changepoint. For illustrative purposes, linear fits were applied before and after each changepoint using the MATLAB function *fitlm*, and R² and slope values of these fits were derived.

The first changepoint analyses focused on whole-brain effects as previous research has identified whole-brain effects surrounding cessations (van Lutterveld et al. 2024). That is, changepoint analyses were first run for the whole-brain average DFA analysis. To explore regional DFA effects, changepoint analysis was also applied to individual EEG channel DFA exponent time series.

#### 2.4.2 Linear mixed effects modeling

As the changepoint analysis does not provide the possibility to directly test against a control condition, linear mixed effects models were applied to whole-brain average and channel-level results to detect differences between timeframes surrounding cessations and the temporal control condition. The R function *lmer* alongside other supporting packages were used (Bates et al., 2015, Kuznetsova et al., 2017, R Core Team, 2024, Wickham et al., 2025, Lenth and Piaskowski, 2026, Wickham and Francois, 2026, Wickham et al., 2026, Wickham and Henry, 2026). For the whole-brain average results, fixed effects were task (control or cessation), epoch number, and a task:epoch interaction term. Random effects included EEG run and an EEG run:channel interaction term. Pairwise epoch comparisons were made using the R function *emmeans* (Lenth and Piaskowski, 2026) with a correction for multiple comparisons using the false discovery rate (FDR; Benjamini & Hochberg, 1995). Statistically significant findings in the whole-brain linear mixed effects analysis were followed up by exploring underlying channel-level DFA results, with linear mixed effects models being applied to channels separately. Fixed effects included task, epoch, and a task:epoch interaction term. EEG run was a random effect. Pairwise epoch comparisons were made again with *emmeans*, but without correction for multiple comparisons given the exploratory nature of this analysis. For both the whole-brain average and channel-level analyses, linear mixed effects models were applied separately to pre-cessation/pre-control marker and post-cessation/post-control marker timeframes.

## 3 Results

### 3.1 Whole-brain DFA

#### 3.1.1 Changepoint analysis

In the pre-cessation timeframe, a 68% probability was observed for one total changepoint, with a 72% probability of that changepoint being at 11.3 s before cessation. For the post-cessation timeframe there was a 77% probability of one total changepoint, with a 97% probability of that changepoint being at 23.6 s after cessation. Illustrative linear fits before and after the observed changepoints revealed a slight decreasing trend from 39.9 s to 11.3 s pre-cessation, and a spike from 11.3 s to 3.1 s pre-cessation. After cessation, the DFA exponent slightly decreased from 3.1 s to 23.6 s post-cessation, where it dropped abruptly and then made a final slight recovery from 23.6 s to 39.9 s post-cessation. No significant pre-control marker or post-control marker changepoints were detected. See Supplementary Material Tables S3 for details regarding changepoint analysis and linear fits for whole-brain average DFA.

#### 3.1.2 Linear mixed effects modeling

**Fig. 2:**
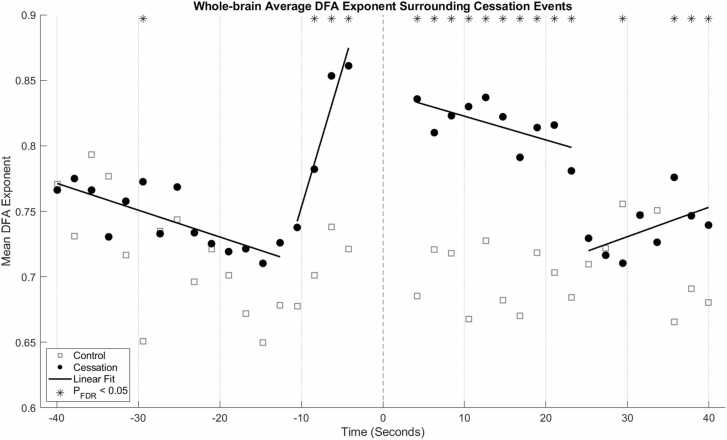
Detrended fluctuation analysis exponent averaged across all 19 channels leading up to and following cessation. The 0 s mark indicates the cessation or control marker. Lines represent linear fits of the time series before and after a changepoint, while asterisks indicate a significant false discovery rate corrected difference between cessation and control at that time point. DFA: detrended fluctuation analysis. FDR: false discovery rate.

Linear mixed effect modeling in the pre-cessation timeframe showed a significant interaction effect of task and epoch (F = 17.0; *p* < 0.001), and follow-up pairwise contrasts showed a significant effect of task after FDR correction for epochs 6 and 16–18 (P_FDR_ values ranging from <0.001–0.03). For the post-cessation timeframe, there was a significant interaction effect of task and epoch (F = 27.8; *p* < 0.001) and follow-up pairwise contrasts showed a significant effect of task after FDR correction for epochs 22–31, 34, and 37–39 (P_FDR_ values ranging from <0.001–0.04). Fig. 2 provides a visual representation of these findings. These results show a modulation of the whole-brain average DFA exponent surrounding cessation events, with a sharp spike pre-cessation and then an elevated period preceding a decrease post-cessation. Consult Supplementary Material Figure S4 for a visual summary of observed whole-brain effects surrounding cessations derived from the studies that have analyzed this case study dataset: the present paper, Chowdhury et al. (2023), and van Lutterveld et al. (2024). Adapting figures from these other studies, this summary displays whole-brain trends in alpha and theta power, functional connectivity, and whole-brain average DFA alongside each other. These results show that the linear effects in criticality observed in the current study are largely independent from the linear observations in the other metrics from the two other studies.

### 3.2 Channel-level DFA

**Fig. 3:**
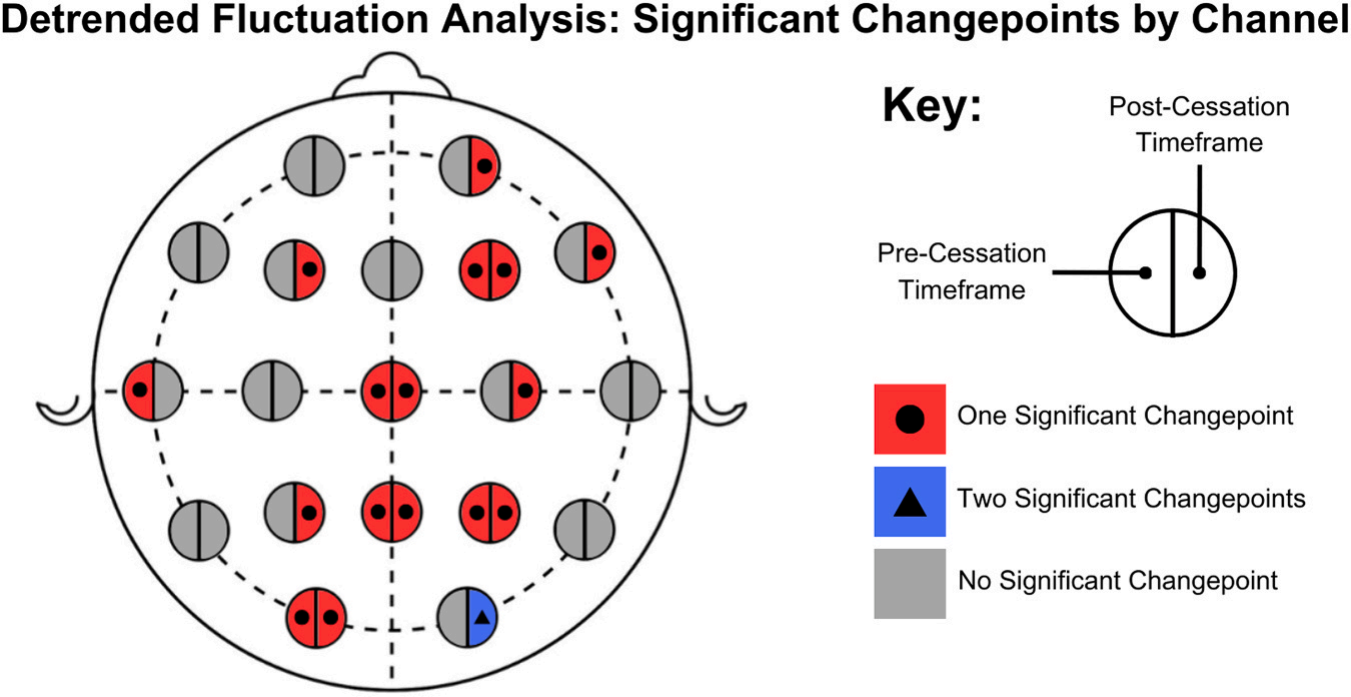
Brain map summary of channel-level detrended fluctuation analysis changepoint analysis. Each circle is one EEG channel, with the left semicircle representing the pre-cessation timeframe and the right semicircle representing the post-cessation timeframe. Red highlighting with a black circle means one significant changepoint was detected, blue highlighting with a black triangle indicates two significant changepoints were identified, and gray highlighting means no significant changepoints were found. Temporal evolution of the DFA exponent for channels with significant changepoints mostly mimics Fig. 2. Most pre-cessation changepoints occur at −11.3 s pre-cessation, and most post-cessation changepoints occur at 23.6 s post-cessation.

**Fig. 4:**
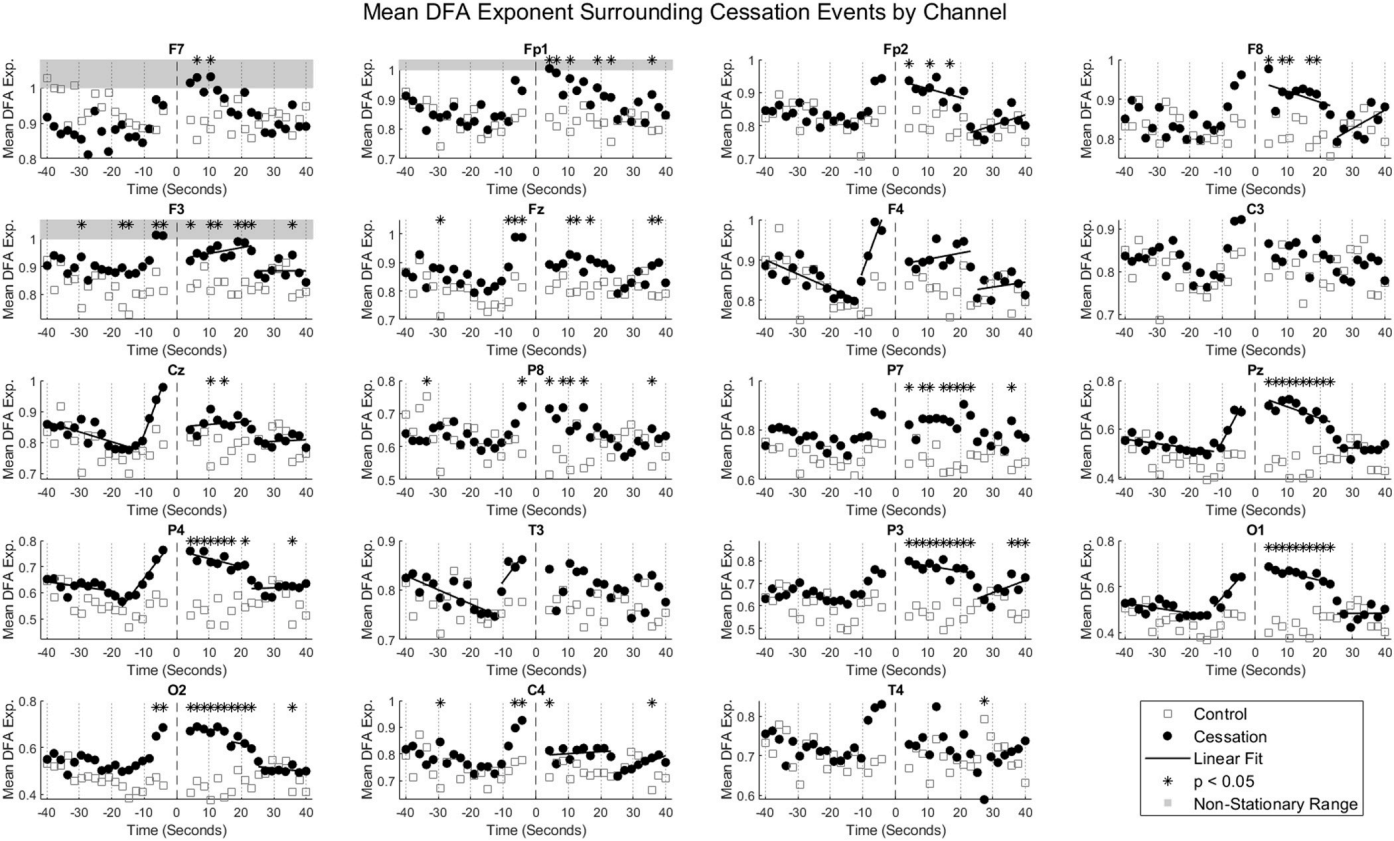
Detrended fluctuation analysis time series by channel. Illustrative linear fits were applied before and after each significant changepoint. Asterisks show where significant differences between cessation and control points were found in linear mixed effects modeling. Gray shading indicates the non-stationary supercritical signal range (i.e., DFA exponent values >1). DFA: detrended fluctuation analysis.

One significant pre-cessation changepoint was detected in 6 of 19 channels (with probabilities of one total changepoint ranging from 54% to 79%, and individual time point changepoint probabilities ranging from 52% to 84%). Four of these six pre-cessation changepoints occurred at 11.3 s pre-cessation, one occurred at 13.3 s pre-cessation, and the final occurred at 17.4 s pre-cessation. One post-cessation changepoint was detected in 10 of 19 channels, and two significant changepoints were detected in 1 additional channel (with probabilities of the total number of changepoints ranging from 53% to 83%, and individual time point changepoint probabilities ranging from 57% to 98%). For the channels with one significant changepoint, 8 of 10 had changepoints at 23.6 s post-cessation, and two had a changepoint at 21.5 s post-cessation. The channel with two significant changepoints had them at 15.4 s and 23.6 s after cessation. No significant pre-control marker or post-control marker changepoints were detected. Fig. 3 provides a graphical representation of channel-level DFA changepoint analysis, and Fig. 4 provides an overview of DFA time series per channel.

Illustrative linear fits applied for EEG channels with significant changepoints followed similar patterns to the whole-brain average DFA exponent. During the pre-cessation timeframe, there was a slight decrease followed by a spike before cessation, while during the post-cessation timeframe the DFA exponent initially decreased slightly or remained stable for most channels, dropped at the changepoint, and then returned towards the general early pre-cessation timeframe baseline level. Channels with a significant pre-cessation changepoint were F4, Cz, Pz, P4, T3, and O1. Channels with significant post-cessation changepoints were Fp2, F8, F3, F4, Cz, Pz, P4, P3, O1, O2, and C4.

For three channels in the frontal and frontopolar regions (i.e., F7, Fp1, F3), around the cessation the mean DFA exponent temporarily exceeded a value of 1, meaning the signal moved just beyond the critical point from stationarity to non-stationarity. For the other channels the average signal moved closer to but not beyond the critical point, or non-stationary threshold. The former channels do not necessarily have a pre-cessation DFA exponent increase greater in magnitude than other channels but rather start at a higher baseline DFA exponent level. Please see Supplementary Material Tables S5 for changepoint analysis results and linear fit information for channel-level DFA.

Exploratory linear mixed effects modeling in the pre-cessation timeframe identified five channels of interest with either a significant main effect of task or interaction of task and epoch: C4, F3, Fz, O2, and P8 (all *p* < 0.05). Follow-up pairwise contrasts for those channels showed between two and five pre-cessation epochs per channel with a significant effect of task (all *p* < 0.05, see Fig. 4). Most of these significant epochs were in the final three epochs before cessation. Post-cessation timeframe modeling showed 16 channels of interest with either a significant main effect of task or interaction of task and epoch: C4, Cz, F3, F7, F8, Fp1, Fp2, Fz, O1, O2, P3, P4, P7, P8, Pz, and T4 (all *p* < 0.05). Follow-up pairwise contrasts for those channels showed between 1 and 13 post-cessation epochs per channel with a significant effect of task (all *p* < 0.05, see Fig. 4). These significant epochs were primarily clustered in the 3.1–23.6 s post cessation as well as the final three epochs. In most channels, a pre-cessation increase in the mean DFA exponent (with or without a significant changepoint) brought the average signal closer to the critical point, while for some frontal and frontopolar channels the increase moved the average signal just into the non-stationary region.

### 3.3 Cessation analysis

We further explored these findings by investigating what happens during and immediately surrounding the cessation itself. For this, the co-occurring artifact needed to be cleaned. We explored several options, finding that applying multi-channel Wiener filtering (MWF) on the three stitched segments from −3.1 s pre-cessation to 3.1 s post-cessation (the three segments that were not analyzed in the main analysis) worked best. MWF is a statistical filtering technique for estimating a signal from noisy measurements and has been shown to be effective in cleaning EEG artifacts (Somers et al., 2018). MWF was performed using the *mwf_getmask* and *mwf_process* functions within MATLAB (Somers et al. 2018), and cleaned data were then filtered in the 1–20 Hz range using the same FFT filter as in the main analysis. All cleaned segments were visually inspected, and six stitched segments were removed due to remaining artifact/noise presence, as well as two segments due to technical difficulties. After un-stitching the segments, 29 of each of the middle three segments were left for further DFA analysis identical to the main analysis. Table 1 shows mean DFA exponent values for the whole-brain and channel-level analyses for the cessation-containing segments and the segments immediately surrounding cessations. Supplementary Figures S6 and S7 show a graphical representation of these findings superimposed on the whole-brain and channel-level −39.9 to −3.1 s pre- and 3.1–39.9 s post-cessation results in Fig. 2 and Fig. 4.

**Table 1:** Mean detrended fluctuation analysis exponent values for the whole-brain and channel-level analyses for segments containing the cessation and segments directly before and after. DFA: Detrended fluctuation analysis.

	**Mean DFA Exponent During and Immediately Surrounding Cessations**		
	**−3.1 to −1.0 s pre-cessation onset**	**−1.0–1.0 s (cessation onset)**	**1.0–3.1 s post-cessation onset**
Whole-brain Average	0.86	0.81	0.96
F7	0.94	0.88	0.98
Fp1	0.93	0.92	0.96
Fp2	0.93	0.91	0.96
F8	0.94	0.90	0.97
F3	0.99	0.95	1.01
Fz	0.97	0.90	0.97
F4	0.92	0.90	0.95
C3	0.97	0.88	0.96
Cz	1.00	0.86	0.96
P8	0.75	0.72	0.87
P7	0.78	0.79	1.01
Pz	0.71	0.65	1.00
P4	0.76	0.72	0.97
T3	0.88	0.85	0.97
P3	0.78	0.75	1.03
O1	0.69	0.62	0.94
O2	0.69	0.62	0.92
C4	0.89	0.81	0.95
T4	0.83	0.77	0.87

Overall, these findings show that the mean DFA exponent in the −3.1 to −1.0 s pre-cessation segment is mostly relatively similar to its preceding −5.1 to −3.1 s segment, followed by a temporary drop during the segment containing cessation onset, which is then followed by an increase at the following 1.0–3.1 s segment. This increase occurs at all channels, which leads the F3, P7, and P3 electrodes to on average incur into supercritical territory. In the whole-brain time series and 11 of 19 electrodes, this segment has the largest mean DFA exponent in the entire −39.9–39.9 s timeframe. This increase is then followed by a drop in the following segment for all channels except F7, Fp1, and F8, and is especially pronounced at F3, Fz and all parietal, central, temporal, and occipital channels.

## 4 Discussion

This is the first study to explore critical dynamics related to advanced meditation cessation events. In a single expert meditator, we confirmed our hypothesis regarding increased DFA exponents surrounding cessations, with a sharp, linear pre-cessation spike in the whole-brain average DFA exponent as well as 6 of 19 individual EEG channels. Moreover, in the whole-brain average and 11 of 19 individual EEG channels, the DFA exponent dropped abruptly and significantly often around 23.6 s post-cessation, after which it returned to the general early pre-cessation timeframe baseline level. Linear mixed effects modeling aligns with findings suggested by BEAST, often demonstrating higher whole-brain average DFA exponent values 11.3–3.1 s prior to cessations as well as in the first approximately 23.6 s of the post-cessation period. The latter was present most prominently in posterior channels. In a few frontal and frontopolar channels, the average DFA exponent crossed over from the stationary to the non-stationary, supercritical range for this advanced meditator. Finally, the segment containing cessation onset was associated with a temporary drop in the mean DFA exponent relative to adjacent segments, followed by an increase in the following segment, which again was followed by a large drop at the next segment for most electrodes.

Pre- and post-cessation timeframe analysis.

Interestingly, a gradient from the frontal to occipital cortex in baseline mean DFA exponent values before the spike was observed, with frontal and frontopolar channels hovering on average closer to criticality. The following approximate baseline pre-cessation mean DFA exponent ranges were observed: frontal and frontopolar from 0.8 to 0.9, central from 0.75 to 0.85, parietal from 0.55 to 0.65, and occipital from 0.5 to 0.6. Because of this anterior to posterior gradient, the pre-cessation DFA exponent increase pushed brain criticality into non-stationary (supercritical) territory for some frontal and frontopolar channels, but only closer to criticality while remaining in stationary territory for all other channels. This suggests that for this meditator, the lead-up to cessations may be associated with activity across the brain becoming less stable and predictable (Kroha and Škoula, 2018). However, it is important to note that in previous applications of DFA in neuroscience, to our knowledge, this particular gradient has not been found (Irrmischer et al., 2018, Thiery et al., 2018, Seleznov et al., 2019). Perhaps it is thus the unique alteration of baseline brain activity during advanced meditation that results in this gradient.

During the post-cessation timeframe in the whole-brain average as well as EEG channels with a significant changepoint, the mean DFA exponent in this single expert meditator often decreased slightly post-cessation for approximately 23 s, after which an abrupt drop was observed. As reported in other literature and confirmed by the length of cessations in the validation button-press run in the current study (median length of 3.6 s; range 2.1 s - 7.2 s) (Chowdhury et al. 2023), cessations generally only last a few seconds (Sayadaw, 1994). Therefore, the approximately 23 s post-cessation strengthened long-range temporal correlations phase cannot be explained by the presence of cessations of the same length, but rather to sustained strengthening of long-range temporal correlations.

Previous assessment of criticality in the brain during focused attention meditation found decreased instead of increased DFA exponents, which correlates to weakened long-range temporal correlations and a movement away from the critical point (Irrmischer et al., 2018, Walter and Hinterberger, 2022a, D’Andrea et al., 2024). The authors argued that a narrowed focus reduces information propagation, thereby bringing about a more subcritical brain state. Interestingly, with cessations we found a movement towards criticality in the lead-up, with an elevated plateau in the early post-cessation period. During cessation setup, various visual phenomena may occur, such as 3D rotating objects or 3D moving landscapes (Chowdhury et al., 2023, van Lutterveld et al., 2024). Moreover, just after cessation, there may be more visual phenomena, such as a lighter purple circle on a darker purple background. The posterior aspect of the brain, where post-cessation differences with the temporal control condition were most pronounced, contains the occipital cortex which processes such visual phenomena. This represents a possible link between phenomenological aspects of cessation events and our neuroscientific findings in this practitioner.

Criticality has also been used to investigate other states of consciousness not related to meditation (Walter and Hinterberger 2022b). For instance, the review offered by Gervais et al. (2023) reports that psychedelics incline the brain closer to the critical point, while in contrast, non-rapid eye movement sleep is associated with more subcritical brain dynamics, and epileptic seizures represent more supercritical brain dynamics. There exist parallels between the subjective experiences reported during psychedelic use and cessations. Like cessations, psychedelics are said to be capable of resetting the brain; Nutt (2019) asserts that psychedelics may disrupt the default mode network and associated depressive processes, thereby potentially restoring the brain to a non-depressive state. Furthermore, anesthesia-induced unconsciousness, like psychedelics, is associated with brain operation closer to the critical point. Thiery et al. (2018) found an increase in long-range temporal correlations with anesthesia-induced unconsciousness compared to wakefulness. They interpret this finding as an increase in signal persistence and regularity, which is associated with decreases in neuronal excitability and cognitive flexibility. Because the same increase in long-range temporal correlations was found surrounding and also during cessation events, these findings may apply to cessations in this single expert meditator as well.

Exploratory cessation timeframe analysis.

Importantly, the segment containing the onset of cessations included the lead up to cessation as well as the start, with the middle point estimated as its onset. This precludes drawing potential inferences about the temporal evolution of criticality during this segment. However, its following segment (1.0–3.1 s post-cessation) contained a marked increase in the mean DFA exponent followed by an immediate drop in the next segment for most channels, which falls within the median length of cessations of 3.6 s in this participant (Chowdhury et al. 2023). This suggests that cessations in this subject may be associated with movement closer to criticality and, for some channels, incurring into supercritical territory. Cessations are often described as a deep reset, leaving the mind feeling refreshed. Being closer to the critical point means generally the brain is more sensitive to perturbations, which aligns with this profound subjective report (Carhart-Harris et al., 2014, Maschke et al., 2023). The presence of some non-stationary EEG signals surrounding cessations during the pre- and post-cessation timeframes, in F7, Fp1, and F3, where the mean DFA exponent exceeded a value of 1, suggests some sort of pronounced shift in mean or variance in the signals. When exploring the segments containing the cessation onset and the segments directly preceding and following, three channels (P7, P3, and F3) reached the supercriticality threshold in the segment after cessation onset. These findings correlate loosely with the dramatic, deep reset experience. Interestingly, in most channels this segment has the largest mean DFA exponent and is often closest to the critical point compared to the rest of the segments, indicating that the brain may be most sensitive to perturbations during cessations in this participant.

Overall, the approximate pattern is a linear increase in criticality leading up to cessation, a temporary drop around the onset of cessation, a large increase during cessation, followed by another drop and temporary sustained elevation before returning to the general early pre-cessation level.

Except for the relatively short period surrounding and during the cessation in some channels, we observed subcritical dynamics during these EEG recordings. This finding is in line with past criticality research which suggest that brains, both human and animal, may as a default operate in a slightly subcritical state (Priesemann, 2014). Finally, Chowdhury et al. (2023) and van Lutterveld et al. (2024) also show linear effects surrounding cessations in the same rare dataset in alpha and theta power and alpha band whole-brain functional interactions respectively (although at different timeframes than the present study), suggesting that neural criticality can provide information beyond these other measures.

### 4.1 Limitations

The present study should be interpreted in light of several limitations. A first limitation constitutes the case-study design, which was utilized as the ability to reach cessations is relatively rare. This design limits generalizability, and the results may only be applicable to cessations of this single expert meditator. Larger studies are needed. Another limitation constitutes the rating of cessations by the meditator. The participant was included in this study because of their extensive and diverse meditation experience as well as their ability to assess the quality of cessations; a fundamental limitation of this study design is the reliance on the subjective grading and report of this one meditator (Chowdhury et al. 2024). However, it is important to note that the grading of cessations, although done by the participant based on their subjective report, is based on criteria that reflects traditional cessation events described in literature (Ñāṇamoli, 2010). Also, the subject’s meditation experiences may be grounded in an interpretation of traditional Therevada practice that differs from that of some others (Anālayo, 2020, Anālayo, 2021), or may be influenced by metaphysical beliefs (Griffiths, 1999). Co-author DMI has published a public, IRB-approved online meditation dataset with video, audio, EEG, detailed phenomenology, and occasionally biometrics (https://osf.io/srfnz/overview). This dataset, which also contains similar events to the present paper, is accessible for exploration by other scientists.

An additional limitation is related to the control condition. It should be noted that the control condition constitutes a temporal control only. That is, as the control segments were derived from the same runs as the cessation segments (although temporally removed from cessations), it can be expected that these segments included cognitive processes related to meditation, and as such do not constitute a fully “pure” control condition. Still, findings from linear mixed effects modeling followed the changepoint analysis surrounding cessations, in which statistical testing was not performed directly versus the control condition. Moreover, there is debate whether cessations are a form of absent consciousness related to mind blanking (Andrillon et al. 2025), although mind blanking is not associated with the beneficial pleasant effects commonly reported after cessations. Moreover, a limitation of the BEAST changepoint analysis toolbox is that by nature of the algorithm, the changepoint probabilities and output model vary slightly each time the software is run on the same data. However, the ability of the algorithm to provide probabilities (the “fuzzy” aspect) was useful. Also, this study is limited by the involuntary eye blink artifact that occurred alongside cessations. This artifact was addressed by removing the middle three EEG segments that contained the blink in the main analysis, as well as additional low-pass frequency filtering to mitigate the influence of any potential anticipatory muscle activity. The additional analysis of the segments containing cessation onset and its immediately surrounding segments is dependent on the accuracy of MWF filtering. Finally and importantly, the present results should be interpreted as exploratory and hypothesis-generating.

## 5 Conclusion

In this study, we provide preliminary evidence for modulation of criticality related to cessation events, with a sharp increase pre-cessation, temporary drop around cessation onset, increase during cessation, and abrupt drop post-cessation. These results add to a growing body of knowledge on advanced meditation and criticality more broadly, suggesting altered critical brain dynamics associated with cessations. Moreover, this study supports the existing concept that critical dynamics differ between and can be used to comment on various states of consciousness.

## CRediT authorship contribution statement

**Sacchet Matthew:** Writing – review & editing, Writing – original draft, Supervision, Project administration, Data curation, Conceptualization. **Lukas van Herk:** Writing – review & editing, Formal analysis. **Ingram Daniel:** Writing – review & editing, Investigation, Conceptualization. **Remko van Lutterveld:** Writing – review & editing, Writing – original draft, Visualization, Supervision, Formal analysis, Data curation, Conceptualization. **Thomas Cahaly:** Writing – review & editing, Writing – original draft, Visualization, Formal analysis, Data curation.

## Compliance with ethical standards

This study was approved by the Western Institutional Review Board (WIRB) and the participant provided informed consent.

## Funding

This work was supported by the 10.13039/100000025National Institute of Mental Health [Project Number R01MH125850]; the Dimension Giving Fund; the Tan Teo Charitable Foundation; and additional individual donors.

## Declaration of Competing Interest

Authors RvL, TC, and LvH declare no conflict of interest. DMI volunteers as Board Chair and Acting CEO of Emergence Benefactors, an international research charity. The fundraising and public relations campaigns of Emergence Benefactors might benefit from the publication of this study. DMI is the author of Mastering the Core Teachings of the Buddha, which is referenced in the Supplementary Material. MDS directs the Meditation Research Program at Massachusetts General Hospital and Harvard Medical School, which has received research funding from DMI/Emergence Benefactors.

## Footnotes

Appendix A Supplementary data associated with this article can be found in the online version at doi:10.1016/j.ibneur.2026.07.011.
