# Supplementary material for: Brain criticality and advanced meditation “cessation” events: An intensively sampled electroencephalography case study
Source: IBRO Neurosci Rep. 2026 Jul 25;21:375–85. doi: 10.1016/j.ibneur.2026.07.011 (PMC13452153; doi:10.1016/j.ibneur.2026.07.011)
Supplement: Supplementary file 1 [file mmc1.docx]

**Supplementary Text S1.** Phenomenology and Cessation Grading Criteria

Please note that because the present paper analyzes the same cessations selected with the same grading criteria, this supplementary text is identical to the grading criteria included in Chowdhury et al. (2023) and van Lutterveld et al. (2024). Cessations were identified, differentiated from other experiences, and graded using the following five cessation components and associated criteria. Major criteria of a given component were required to meet a given grade. Minor criteria of a given component supported the classification of major criteria but were not necessary to satisfy meeting criteria for a given component.

1. Setup
*Major:*
1. Clear progression through the entire Stages of Insight (Nanamoli 2010).
*Minor:*
1. Experience of formlessness within minutes prior to the Cessation.
2. Awareness of all of experience beginning to synchronize in “gently rocking” manner.
3. Awareness of entering into “3D” visual experiences or other visual phenomena that tend to precede Cessations, such as vivid 3D moving landscapes or 3D rotating objects.

2. Entrance:
*Major:*
1. Experiential clarity of Three Door phenomenology. This major criterion includes six basic presentations that are combinations of the three elements of: (1) the collapse of the sense of object and subject (i.e., no-self), (2) the sense of the complete and somewhat jarring separation of experience from the sense of awareness (suffering), and (3) the complete pulsing of all experience (impermanence). A simplified explanation of the six presentations is: (1) the No Self/Impermanence Door is the collapse of subject and object and is perceived in 3-4 complete pulses of all of experience (2) The Suffering/Impermanence Door is the removal of experience from awareness and is perceived in 3-4 pulses of all of experience, and (3) the No-Self/Suffering Door, in which experience rotates, subject and object appear to trade places, and experience vanishes when this is complete. Each of these doors may be reversed if one or the other characteristic is more predominant, yielding the Impermanence/No-self Door, the Impermanence/Suffering Door, and the Suffering/No-Self Door. There are other more rare phenomenological variants of Three Door phenomenology and they follow similar themes, and all appear to have the same outcome. These Doors tend to take approximately 100 to 300 ms as estimated experientially. Each Door involves the phenomenological sense of involving all of experience, with the sense of complete, totally inclusive, undivided, and synchronized attention that is called Conformity Knowledge (Ingram 2018).
2. Occurrence of fluttering eyelids.

3. Event:
*Major:*
Experience entirely gone for the cessation event. A clean sense of the discontinuity of experience based on the memories of the sensations before and after cessation and their disjoined relationship.

4. Exit:
*Major:*
1. Mind experienced as “freshly restarting”, having been “deeply reset”. Both body and mind feeling refreshed, satisfying completion, and clean in a way that only happens after cessations.
2. Pattern of breathing changed in the predictable way such that there was a sense of breathing starting on the inbreath and moving in a deeper, more fluid way immediately afterwards.
3. Experience did not have “analogue power-down then power-up” characteristic of Nirodha Samapatti, which involves the rapid, smooth (non-strobing, thus analogue (rather than discrete/digital)), complete cessation of consciousness of internal verbal formations, bodily sensations, and then feeling and perception itself (Nanamoli 2010), with the exit occurring in the reverse order and taking approximately the same amount of time.

5. Afterglow/Aftereffects:
*Major:*
1. Experience of “afterglow” over seconds to minutes of increasing rapture, refreshment, and mental cleanliness.
2. Afterglow was not like the deeper, stronger, and longer-lasting afterglow of Nirodha Samapatti.
*Minor:*
1. Experience of visuals routinely noticed immediately after cessations in this particular practitioner (e.g. a lighter purple circle on a darker purple background).
2. Mind re-inclined toward additional cessations.
3. Mind returned to the beginning of a cycle of the Stages of Insight.

*Grades:*
A. If all five components (and all major criteria therein) were met with a high degree of certainty, the given cessation was classified as Grade A.
B. If four of the five components were met with a high degree of certainty, but one component was met with a moderate degree of certainty, the given cessation was classified as Grade B. Often, Grade B cessations did not meet Grade A classification because they did not include perception of the Entrance with a high degree of certainty that it met the phenomenology of the Three Doors.
C. If there is still belief that a cessation may have occurred, but the phenomenology was not clear enough to meet criteria for Grades A or B, then the given experience was classified as Grace C. This may occur when the Exit and Afterglow/After-effects were clear, but the Entrance was not, in addition to other features not having been clear. For example, uncertainty regarding the completeness of the experiential discontinuity (The Event), the Event occurred early in the Setup without clear final Stages of Insight (e.g., Equanimity), or the Entrance Door was not clearly perceived.
D. If the Exit and Afterglow criteria were not met, the given experience was not considered for further evaluation and was thus not graded as a cessation.

**Supplementary Text S2.** Detrended Fluctuation Analysis Procedure

As detailed by Bryce and Sprague (2012), Detrended Fluctuation Analysis (DFA) follows the general procedure outlined below:

1. Convert the time series into a mean-centered cumulative sum by the following formula:

$y(t)=\sum_{i=1}^{t} [x(i)-\left\langle x \right\rangle]$ , where $\left\langle x \right\rangle$ is the average value of the time series.

2. Divide data into boxes of different sizes $\Delta n$ that are ideally equally spaced on a logarithmic scale. The minimum box size was above 4 samples (Hardstone et al. 2012; Kuznetsov and Rhea 2017).
3. Determine the least squares straight-line fit $y_{\Delta n}(t)$ for one box.
4. Compute the root-mean-squared residual $F(\Delta n)$ for one box by the following formula:

$F(\Delta n)=\sqrt{\frac{1}{N}\sum_{t=1}^{N} {[y(t)-y_{\Delta n}(t)]}^{2}}$ , where $N$ is the total number of values in the box.

5. Repeat steps 3 and 4 for the different box sizes.
6. Construct a log-log plot of $F(\Delta n)$ vs $\Delta n$.
7. Apply a linear regression to the log-log plot; the slope is the DFA exponent $\alpha$.

**Supplementary Tables S3.** Detrended Fluctuation Analysis (Whole-brain Average)

*For readability of tables, all time values were rounded to the nearest tenth of a second. Segments are 2.048 seconds in duration. Additionally, segment numbers, not time values, are listed for changepoint analysis; there are 18 pre-cessation or pre-control marker segments, and 18 post-cessation or post-control marker segments.*

**Table S3.1.** Whole-brain Average DFA Changepoint Analysis

**Note that for post-cessation and post-control marker time series, the segment number represents the timeframe assuming the first post-cessation or post-control marker timeframe is segment 1.*

†*Depending on the time series, the changepoint analysis output highlighted either one individual changepoint probability or two individual changepoint probabilities*.

*Significant changepoints (both probabilities>0.50) are bolded.*

*CP: Changepoint. NCP: Number of changepoints.*

|  | Probability of X Number of Changepoints | | | |  | Which Segment is Changepoint? | | | |
| --- | --- | --- | --- | --- | --- | --- | --- | --- | --- |
|  | 0 | 1 | 2 | 3 | NCP Mean | CP 1 | Probability | CP 2 | Probability |
| Pre-Control Marker | 0.429 | 0.474 | 0.093 | 0.04 | 0.67 | 6 | 0.323 | 13 | 0.222 |
| Post-Control Marker* | 0.520 | 0.408 | 0.068 | 0.003 | 0.55 | 15 | 0.287 | † | † |
| Pre-Cessation | 0.166 | **0.681** | 0.150 | 0.003 | 0.99 | **15** | **0.721** | † | † |
| Post-Cessation* | 0.020 | **0.773** | 0.188 | 0.019 | 1.21 | **11** | **0.971** | 7 | 0.165 |

**Table S3.2.** Whole-brain Average DFA Illustrative Linear Fit Information

*Timeframes are bisected by a significant changepoint.*

*DE: Detrended fluctuation analysis exponent.*

|  | **Time Interval (s)** | **R^2^** | **Slope (DE/s)** |
| --- | --- | --- | --- |
| ***Pre-Cessation Timeframe 1*** | -39.9 to -11.3 | 0.61 | -0.002 |
| ***Pre-Cessation Timeframe 2*** | -11.3 to -3.1 | 0.93 | 0.02 |
| ***Post-Cessation Timeframe 1*** | 3.1 to 23.6 | 0.40 | -0.002 |
| ***Post-Cessation Timeframe 2*** | 23.6 to 39.9 | 0.31 | 0.002 |

**Table S3.3.** Segment Whole-brain Average DFA Exponent Values

*DE: Detrended fluctuation analysis exponent.*

| **Time (s)** | **Control DE** | **Cessation DE** |
| --- | --- | --- |
| -39.9 to -37.9 | 0.77 | 0.77 |
| -37.9 to -35.8 | 0.73 | 0.77 |
| -35.8 to -33.8 | 0.79 | 0.77 |
| -33.8 to -31.7 | 0.78 | 0.73 |
| -31.7 to -29.7 | 0.72 | 0.76 |
| -29.7 to -27.7 | 0.65 | 0.77 |
| -27.7 to -25.6 | 0.73 | 0.73 |
| -25.6 to -23.6 | 0.74 | 0.77 |
| -23.6 to -21.5 | 0.70 | 0.73 |
| -21.5 to -19.5 | 0.72 | 0.73 |
| -19.5 to -17.4 | 0.70 | 0.72 |
| -17.4 to -15.4 | 0.67 | 0.72 |
| -15.4 to -13.3 | 0.65 | 0.71 |
| -13.3 to -11.3 | 0.68 | 0.73 |
| -11.3 to -9.2 | 0.68 | 0.74 |
| -9.2 to -7.2 | 0.70 | 0.78 |
| -7.2 to -5.1 | 0.74 | 0.85 |
| -5.1 to -3.1 | 0.72 | 0.86 |
| 3.1 to 5.1 | 0.69 | 0.84 |
| 5.1 to 7.2 | 0.72 | 0.81 |
| 7.2 to 9.2 | 0.72 | 0.82 |
| 9.2 to 11.3 | 0.67 | 0.83 |
| 11.3 to 13.3 | 0.73 | 0.84 |
| 13.3 to 15.4 | 0.68 | 0.82 |
| 15.4 to 17.4 | 0.67 | 0.79 |
| 17.4 to 19.5 | 0.72 | 0.81 |
| 19.5 to 21.5 | 0.70 | 0.82 |
| 21.5 to 23.6 | 0.68 | 0.78 |
| 23.6 to 25.6 | 0.71 | 0.73 |
| 25.6 to 27.6 | 0.72 | 0.72 |
| 27.6 to 29.7 | 0.76 | 0.71 |
| 29.7 to 31.7 | 0.75 | 0.75 |
| 31.7 to 33.8 | 0.75 | 0.73 |
| 33.8 to 35.8 | 0.67 | 0.78 |
| 35.8 to 37.9 | 0.69 | 0.75 |
| 37.9 to 39.9 | 0.68 | 0.74 |

**Supplementary Figure S4.** Compiled Cessation Results From Multiple Studies

*Summary of whole-brain effects surrounding cessations from the present paper and previous studies on this dataset. (a) Whole-brain average DFA exponent trends surrounding cessations from the present paper, where linear regressions are plotted before and after significant changepoints. (b) Theta band power differences (cessation-control), with a significant pre-cessation linear trend marked by a line. Adapted from Chowdhury et al. (2023) with use of plotidigitzer.com. (c) Alpha band power differences (cessation-control), with significant pre- and post-cessation linear trends marked by lines. Adapted from Chowdhury et al. (2023) with use of plotdigitizer.com. (d) Average phase lag index, with significant regressions marked by lines. Adapted from van Lutterveld et al. (2024).*

**Supplementary Tables S5.** Detrended Fluctuation Analysis (Channel-Level)

*For readability of tables, all time values were rounded to the nearest tenth of a second. Segments are 2.048 seconds in duration. Additionally, segment numbers, not time values, are listed for changepoint analysis; there are 18 pre-cessation or pre-control marker segments, and 18 post-cessation or post-control marker segments.*

**Table S5.1.** Channel-Level DFA Changepoint Analysis (Pre-Cessation Timeframe)

†*Depending on the time series, the changepoint analysis output highlighted either one individual changepoint probability or two individual changepoint probabilities*.

*Significant changepoints (both probabilities>0.50) are bolded.*

*CP: Changepoint. NCP: Number of changepoints.*

|  | Probability of X Number of Changepoints | | | |  | Which Segment is Changepoint? | | | |
| --- | --- | --- | --- | --- | --- | --- | --- | --- | --- |
|  | 0 | 1 | 2 | 3 | NCP Mean | CP 1 | Probability | CP 2 | Probability |
| **F7** | 0.520 | 0.411 | 0.066 | 0.003 | 0.55 | 10 | 0.275 | † | † |
| **Fp1** | 0.508 | 0.417 | 0.070 | 0.004 | 0.57 | 10 | 0.218 | † | † |
| **Fp2** | 0.428 | 0.476 | 0.093 | 0.003 | 0.67 | 15 | 0.315 | † | † |
| **F8** | 0.449 | 0.468 | 0.080 | 0.003 | 0.64 | 15 | 0.279 | † | † |
| **F3** | 0.437 | 0.469 | 0.091 | 0.003 | 0.66 | 7 | 0.282 | 15 | 0.248 |
| **Fz** | 0.463 | 0.457 | 0.077 | 0.004 | 0.62 | 15 | 0.262 | † | † |
| **F4** | 0.273 | **0.572** | 0.147 | 0.008 | 0.89 | **15** | **0.530** | † | † |
| **C3** | 0.481 | 0.443 | 0.073 | 0.003 | 0.60 | 11 | 0.315 | † | † |
| **Cz** | 0.165 | **0.655** | 0.174 | 0.006 | 1.02 | **15** | **0.677** | † | † |
| **P8** | 0.507 | 0.419 | 0.070 | 0.004 | 0.57 | 9 | 0.223 | † | † |
| **P7** | 0.460 | 0.442 | 0.092 | 0.005 | 0.64 | 10 | 0.295 | † | † |
| **Pz** | 0.135 | **0.757** | 0.106 | 0.002 | 0.98 | **15** | **0.700** | † | † |
| **P4** | 0.045 | **0.788** | 0.162 | 0.005 | 1.13 | **12** | **0.844** | 5 | 0.101 |
| **T3** | 0.299 | **0.543** | 0.135 | 0.023 | 0.88 | **15** | **0.521** | † | † |
| **P3** | 0.305 | 0.554 | 0.134 | 0.007 | 0.84 | 15 | 0.414 | † | † |
| **O1** | 0.058 | **0.780** | 0.158 | 0.005 | 1.11 | **14** | **0.650** | † | † |
| **O2** | 0.363 | 0.546 | 0.089 | 0.003 | 0.73 | 15 | 0.406 | † | † |
| **C4** | 0.345 | 0.549 | 0.102 | 0.005 | 0.77 | 15 | 0.431 | † | † |
| **T4** | 0.390 | 0.527 | 0.079 | 0.004 | 0.70 | 15 | 0.344 | † | † |

**Table S5.2.** Channel-Level DFA Illustrative Linear Fit Information (Pre-Cessation Timeframe)

*Timeframes are bisected by a significant changepoint.*

†*Only channels with a significant pre-cessation changepoint had illustrative linear fits applied.*

*DE: Detrended fluctuation analysis exponent.*

|  | ***Pre-Cessation Timeframe 1*** | | | ***Pre-Cessation Timeframe 2*** | | |
| --- | --- | --- | --- | --- | --- | --- |
|  | **Time Interval (s)** | **R^2^** | **Slope (DE/s)** | **Time Interval (s)** | **R^2^** | **Slope (DE/s)** |
| **F7** | † | † | † | † | † | † |
| **Fp1** | † | † | † | † | † | † |
| **Fp2** | † | † | † | † | † | † |
| **F8** | † | † | † | † | † | † |
| **F3** | † | † | † | † | † | † |
| **Fz** | † | † | † | † | † | † |
| **F4** | -39.9 to -11.3 | 0.63 | -0.004 | -11.3 to -3.1 | 0.81 | 0.02 |
| **C3** | † | † | † | † | † | † |
| **Cz** | -39.9 to -11.3 | 0.60 | -0.003 | -11.3 to -3.1 | 0.99 | 0.03 |
| **P8** | † | † | † | † | † | † |
| **P7** | † | † | † | † | † | † |
| **Pz** | -39.9 to -11.3 | 0.36 | -0.002 | -11.3 to -3.1 | 0.87 | 0.03 |
| **P4** | -39.9 to -17.4 | 0.26 | -0.002 | -17.4 to -3.1 | 0.95 | 0.02 |
| **T3** | -39.9 to -11.3 | 0.65 | -0.003 | -11.3 to -3.1 | 0.62 | 0.009 |
| **P3** | † | † | † | † | † | † |
| **O1** | -39.9 to -13.3 | 0.44 | -0.002 | -13.3 to -3.1 | 0.78 | 0.02 |
| **O2** | † | † | † | † | † | † |
| **C4** | † | † | † | † | † | † |
| **T4** | † | † | † | † | † | † |

**Table S5.3.** Channel-Level DFA Changepoint Analysis (Post-Cessation Timeframe)

†*Depending on the time series, the changepoint analysis output highlighted either one individual changepoint probability or two individual changepoint probabilities*.

*Note that for the post-cessation time series, the segment number represents the timeframe assuming the first post-cessation timeframe is segment 1.* *Significant changepoints (both probabilities>0.50) are bolded.*

*CP: Changepoint. NCP: Number of changepoints.*

|  | Probability of X Number of Changepoints | | | |  | Which Segment is Changepoint? | | | |
| --- | --- | --- | --- | --- | --- | --- | --- | --- | --- |
|  | 0 | 1 | 2 | 3 | NCP Mean | CP 1 | Probability | CP 2 | Probability |
| **F7** | 0.327 | 0.484 | 0.185 | 0.004 | 0.87 | 12 | 0.474 | 6 | 0.284 |
| **Fp1** | 0.397 | 0.524 | 0.076 | 0.003 | 0.69 | 11 | 0.395 | † | † |
| **Fp2** | 0.087 | **0.610** | 0.225 | 0.078 | 1.29 | **10** | **0.846** | 14 | 0.286 |
| **F8** | 0.277 | **0.628** | 0.090 | 0.005 | 0.82 | **11** | **0.585** | † | † |
| **F3** | 0.235 | **0.674** | 0.087 | 0.004 | 0.86 | **11** | **0.644** | 5 | 0.137 |
| **Fz** | 0.353 | 0.554 | 0.088 | 0.004 | 0.74 | 11 | 0.499 | † | † |
| **F4** | 0.143 | **0.705** | 0.145 | 0.008 | 1.02 | **11** | **0.782** | 5 | 0.177 |
| **C3** | 0.496 | 0.434 | 0.067 | 0.003 | 0.58 | 8 | 0.257 | † | † |
| **Cz** | 0.193 | **0.730** | 0.074 | 0.002 | 0.89 | **11** | **0.688** | † | † |
| **P8** | 0.227 | 0.685 | 0.083 | 0.005 | 0.87 | 7 | 0.433 | † | † |
| **P7** | 0.464 | 0.461 | 0.071 | 0.004 | 0.62 | 11 | 0.307 | † | † |
| **Pz** | 0.075 | **0.565** | 0.349 | 0.011 | 1.30 | **11** | **0.875** | 6 | 0.342 |
| **P4** | 0.024 | **0.826** | 0.144 | 0.007 | 1.13 | **10** | **0.941** | 5 | 0.129 |
| **T3** | 0.512 | 0.413 | 0.072 | 0.003 | 0.57 | 15 | 0.283 | † | † |
| **P3** | 0.170 | **0.675** | 0.138 | 0.016 | 1.00 | **11** | **0.715** | † | † |
| **O1** | 0.011 | **0.772** | 0.211 | 0.005 | 1.21 | **11** | **0.983** | 7 | 0.203 |
| **O2** | 0.017 | 0.412 | **0.561** | 0.010 | 1.56 | **11** | **0.976** | **7** | **0.569** |
| **C4** | 0.253 | **0.529** | 0.187 | 0.031 | 1.00 | **11** | **0.649** | † | † |
| **T4** | 0.490 | 0.435 | 0.072 | 0.004 | 0.59 | 11 | 0.284 | 5 | 0.205 |

**Table S5.4.** Channel-Level DFA Illustrative Linear Fit Information (Post-Cessation Timeframe)

**Channel O2 only (two significant post-cessation changepoints).*

†*Only channels with a significant post-cessation changepoint had illustrative linear fits applied.*

*Timeframes are separated by significant changepoint(s).*

*DE: Detrended fluctuation analysis exponent.*

|  | ***Post-Cessation Timeframe 1*** | | | ***Post-Cessation Timeframe 2*** | | | ***Post-Cessation Timeframe 3**** | | |
| --- | --- | --- | --- | --- | --- | --- | --- | --- | --- |
|  | **Time Interval (s)** | **R^2^** | **Slope (DE/s)** | **Time Interval (s)** | **R^2^** | **Slope (DE/s)** | **Time Interval (s)** | **R^2^** | **Slope (DE/s)** |
| **F7** | † | † | † | † | † | † | † | † | † |
| **Fp1** | † | † | † | † | † | † | † | † | † |
| **Fp2** | 3.1 to 21.5 | 0.29 | -0.003 | 21.5 to 39.9 | 0.29 | 0.003 | N/A | N/A | N/A |
| **F8** | 3.1 to 23.6 | 0.28 | -0.003 | 23.6 to 39.9 | 0.40 | 0.005 | N/A | N/A | N/A |
| **F3** | 3.1 to 23.6 | 0.35 | 0.002 | 23.6 to 39.9 | 6e-6 | -2e-5 | N/A | N/A | N/A |
| **Fz** | † | † | † | † | † | † | † | † | † |
| **F4** | 3.1 to 23.6 | 0.12 | 0.002 | 23.6 to 39.9 | 0.06 | 0.001 | N/A | N/A | N/A |
| **C3** | † | † | † | † | † | † | † | † | † |
| **Cz** | 3.1 to 23.6 | 0.05 | 0.0008 | 23.6 to 39.9 | 0.03 | 0.0006 | N/A | N/A | N/A |
| **P8** | † | † | † | † | † | † | † | † | † |
| **P7** | † | † | † | † | † | † | † | † | † |
| **Pz** | 3.1 to 23.6 | 0.58 | -0.005 | 23.6 to 39.9 | 0.007 | -0.0004 | N/A | N/A | N/A |
| **P4** | 3.1 to 21.5 | 0.52 | -0.003 | 21.5 to 39.9 | 0.01 | 0.0004 | N/A | N/A | N/A |
| **T3** | † | † | † | † | † | † | † | † | † |
| **P3** | 3.1 to 23.6 | 0.31 | -0.002 | 23.6 to 39.9 | 0.35 | 0.006 | N/A | N/A | N/A |
| **O1** | 3.1 to 23.6 | 0.66 | -0.004 | 23.6 to 39.9 | 5e-5 | 5e-5 | N/A | N/A | N/A |
| **O2** | 3.1 to 15.4 | 0.01 | -0.0003 | 15.4 to 23.6 | 0.11 | -0.003 | 23.6 to 39.9 | 0.19 | -0.001 |
| **C4** | 3.1 to 23.6 | 0.05 | 0.0008 | 23.6 to 39.9 | 0.77 | 0.005 | N/A | N/A | N/A |
| **T4** | † | † | † | † | † | † | † | † | † |

**Table S5.5.** Channel-Level DFA Changepoint Analysis (Pre-Control Marker Timeframe)

†*Depending on the time series, the changepoint analysis output highlighted either one individual changepoint probability or two individual changepoint probabilities*.

*CP: Changepoint. NCP: Number of changepoints.*

|  | Probability of X Number of Changepoints | | | |  | Which Segment is Changepoint? | | | |
| --- | --- | --- | --- | --- | --- | --- | --- | --- | --- |
|  | 0 | 1 | 2 | 3 | NCP Mean | CP 1 | Probability | CP 2 | Probability |
| **F7** | 0.520 | 0.411 | 0.067 | 0.002 | 0.55 | 11 | 0.250 | 6 | 0.184 |
| **Fp1** | 0.444 | 0.469 | 0.084 | 0.003 | 0.65 | 6 | 0.299 | 12 | 0.244 |
| **Fp2** | 0.498 | 0.424 | 0.074 | 0.004 | 0.58 | 15 | 0.298 | † | † |
| **F8** | 0.518 | 0.411 | 0.069 | 0.002 | 0.56 | 7 | 0.285 | † | † |
| **F3** | 0.490 | 0.433 | 0.075 | 0.003 | 0.59 | 6 | 0.247 | 13 | 0.229 |
| **Fz** | 0.444 | 0.467 | 0.086 | 0.003 | 0.65 | 6 | 0.370 | † | † |
| **F4** | 0.337 | 0.535 | 0.122 | 0.006 | 0.80 | 6 | 0.469 | † | † |
| **C3** | 0.487 | 0.430 | 0.079 | 0.003 | 0.60 | 6 | 0.316 | † | † |
| **Cz** | 0.456 | 0.458 | 0.082 | 0.004 | 0.63 | 6 | 0.286 | 13 | 0.215 |
| **P8** | 0.454 | 0.457 | 0.085 | 0.003 | 0.64 | 6 | 0.304 | 13 | 0.223 |
| **P7** | 0.471 | 0.432 | 0.092 | 0.006 | 0.63 | 6 | 0.266 | 14 | 0.225 |
| **Pz** | 0.431 | 0.452 | 0.111 | 0.007 | 0.69 | 13 | 0.286 | 5 | 0.272 |
| **P4** | 0.410 | 0.470 | 0.113 | 0.007 | 0.72 | 5 | 0.290 | 13 | 0.282 |
| **T3** | 0.527 | 0.405 | 0.065 | 0.003 | 0.54 | 6 | 0.274 | † | † |
| **P3** | 0.502 | 0.419 | 0.074 | 0.005 | 0.58 | 12 | 0.283 | † | † |
| **O1** | 0.457 | 0.430 | 0.099 | 0.015 | 0.67 | 13 | 0.306 | 5 | 0.209 |
| **O2** | 0.150 | 0.416 | 0.417 | 0.018 | 1.30 | 5 | 0.617 | 13 | 0.532 |
| **C4** | 0.501 | 0.422 | 0.073 | 0.003 | 0.58 | 6 | 0.233 | 13 | 0.226 |
| **T4** | 0.475 | 0.442 | 0.080 | 0.004 | 0.61 | 6 | 0.325 | † | † |

**Table S5.6.** Channel-Level DFA Changepoint Analysis (Post-Control Marker Timeframe)

†*Depending on the time series, the changepoint analysis output highlighted either one individual changepoint probability or two individual changepoint probabilities*.

*Note that for the post-control marker time series, the segment number represents the timeframe assuming the first post-control marker timeframe is segment 1.*

*CP: Changepoint. NCP: Number of changepoints.*

|  | Probability of X Number of Changepoints | | | |  | Which Segment is Changepoint? | | | |
| --- | --- | --- | --- | --- | --- | --- | --- | --- | --- |
|  | 0 | 1 | 2 | 3 | NCP Mean | CP 1 | Probability | CP 2 | Probability |
| **F7** | 0.524 | 0.411 | 0.062 | 0.003 | 0.54 | 10 | 0.240 | 0 | 0 |
| **Fp1** | 0.523 | 0.407 | 0.068 | 0.003 | 0.55 | 10 | 0.238 | 0 | 0 |
| **Fp2** | 0.515 | 0.412 | 0.069 | 0.003 | 0.56 | 6 | 0.244 | 0 | 0 |
| **F8** | 0.511 | 0.413 | 0.071 | 0.005 | 0.57 | 10 | 0.242 | 0 | 0 |
| **F3** | 0.523 | 0.405 | 0.069 | 0.003 | 0.55 | 13 | 0.283 | 0 | 0 |
| **Fz** | 0.519 | 0.402 | 0.074 | 0.004 | 0.56 | 15 | 0.289 | 0 | 0 |
| **F4** | 0.516 | 0.417 | 0.064 | 0.003 | 0.55 | 7 | 0.284 | 0 | 0 |
| **C3** | 0.517 | 0.408 | 0.071 | 0.003 | 0.56 | 6 | 0.218 | 15 | 0.216 |
| **Cz** | 0.518 | 0.407 | 0.072 | 0.003 | 0.56 | 14 | 0.286 | 0 | 0 |
| **P8** | 0.484 | 0.433 | 0.079 | 0.005 | 0.60 | 5 | 0.313 | 0 | 0 |
| **P7** | 0.518 | 0.409 | 0.069 | 0.004 | 0.56 | 14 | 0.281 | 0 | 0 |
| **Pz** | 0.505 | 0.421 | 0.072 | 0.003 | 0.57 | 13 | 0.241 | 6 | 0.207 |
| **P4** | 0.506 | 0.422 | 0.070 | 0.003 | 0.57 | 13 | 0.243 | 6 | 0.202 |
| **T3** | 0.519 | 0.406 | 0.071 | 0.003 | 0.56 | 15 | 0.219 | 6 | 0.213 |
| **P3** | 0.507 | 0.417 | 0.073 | 0.003 | 0.57 | 13 | 0.245 | 6 | 0.202 |
| **O1** | 0.464 | 0.449 | 0.085 | 0.003 | 0.63 | 7 | 0.288 | 14 | 0.222 |
| **O2** | 0.491 | 0.430 | 0.075 | 0.004 | 0.59 | 13 | 0.288 | 0 | 0 |
| **C4** | 0.521 | 0.409 | 0.067 | 0.003 | 0.55 | 13 | 0.282 | 0 | 0 |
| **T4** | 0.514 | 0.421 | 0.063 | 0.002 | 0.55 | 12 | 0.292 | 0 | 0 |

**Supplementary Figure S6.** Whole-brain Average DFA Surrounding and Including Cessation Events

*Detrended fluctuation analysis exponent averaged across all 19 channels leading up to, including, and following cessation. Points indicated by a red triangle represent the segments directly before, containing, and directly after cessation. The 0 s mark indicates the cessation or control marker. DFA: detrended fluctuation analysis.*

**Supplementary Figure S7.** Mean DFA Exponent Surrounding and Including Cessation Events by Channel

*Detrended fluctuation analysis time series by channel leading up to, including, and following cessation. Points indicated by a red triangle represent the segments directly before, containing, and directly after cessation. Gray shading indicates the non-stationary supercritical signal range (i.e., DFA exponent values >1). DFA: detrended fluctuation analysis.*

**Supplementary References**

Bryce RM, Sprague KB. 2012. Revisiting detrended fluctuation analysis. Sci Rep. 2(1):315. https://doi.org/10.1038/srep00315

Chowdhury A et al. 2023. Investigation of advanced mindfulness meditation “cessation” experiences using EEG spectral analysis in an intensively sampled case study. Neuropsychologia. 190:108694. https://doi.org/10.1016/j.neuropsychologia.2023.108694

Hardstone R et al. 2012. Detrended Fluctuation Analysis: A Scale-Free View on Neuronal Oscillations. Front Physio. 3. http://journal.frontiersin.org/article/10.3389/fphys.2012.00450/abstract. https://doi.org/10.3389/fphys.2012.00450

Ingram DM. 2018. Mastering the core teachings of the Buddha: an unusually hardcore Dharma book. Revised and expanded edition. Aeon.

Kuznetsov NA, Rhea CK. 2017. Power considerations for the application of detrended fluctuation analysis in gait variability studies Podobnik B, editor. PLoS ONE. 12(3):e0174144. https://doi.org/10.1371/journal.pone.0174144

van Lutterveld R, Chowdhury A, Ingram DM, Sacchet MD. 2024. Neurophenomenological Investigation of Mindfulness Meditation “Cessation” Experiences Using EEG Network Analysis in an Intensively Sampled Adept Meditator. Brain Topogr. [published online ahead of print]. https://doi.org/10.1007/s10548-024-01052-4

Nanamoli B. 2010. Visuddhimagga: the path of purification. Buddhist Publication Society.
